# Acquired hemophilia as the cause of life-threatening hemorrhage in a 94-year-old man: a case report

**DOI:** 10.1186/1752-1947-4-231

**Published:** 2010-07-29

**Authors:** Theodoros Kelesidis, Jonelle Raphael, Elizabeth Blanchard, Rekha Parameswaran

**Affiliations:** 1 Department of Medicine, Caritas St Elizabeth's Medical Center, Tufts University School of Medicine, Boston, MA, USA

## Abstract

**Introduction:**

Acquired factor VIII deficiency is a rare entity that can lead to severe and life-threatening bleeding. We describe a case of severe bleeding from the tongue secondary to acquired hemophilia and discuss treatment options, including aminocaproic acid and recombinant factor VIII, which have not been widely reported in the literature for the management of such patients.

**Case presentation:**

A 94-year-old Caucasian man presented to our institution with diffuse bruising and extensive bleeding from the tongue secondary to mechanical trauma. He had no prior history of bleeding and his medical history was unremarkable except for dementia and hypertension. Coagulation studies revealed a prolonged activated partial thromboplastin time and a mixing study was consistent with the presence of an inhibitor. Quantitative assays revealed a reduced level of factor VIII activity (1%) and the presence of a factor VIII inhibitor, measured at seven Bethesda units, in the serum. Oral prednisone therapy (60mg/day) was given. He also received intravenous aminocaproic acid and human concentrate of factor VIII (Humate-P) and topical anti-thrombolytic agents (100 units of topical thrombin cream). His hospital course was prolonged because of persistent bleeding and the development of profuse melena. He required eight units of packed red blood cells for transfusion. Hospitalization was also complicated by bradycardia of unclear etiology, which started after infusion of aminocaproic acid. His activated partial thromboplastin time gradually normalized. He was discharged to a rehabilitation facility three weeks later with improving symptoms, stable hematocrit and resolving bruises.

**Conclusions:**

Clinicians should suspect a diagnosis of acquired hemophilia in older patients with unexplained persistent and profound bleeding from uncommon soft tissues, including the tongue. Use of factor VIII (Humate-P) and aminocaproic acid can be useful in this coagulopathy but clinicians should be aware of possible life-threatening side effects in older patients, including bradycardia.

## Introduction

Acquired hemophilia A is defined as the development of factor VIII inhibitors in a patient who was previously non-hemophilic. The inhibitors can develop in association with autoimmune disease, allergic drug reactions, malignancies, and pregnancy [1]. The incidence of acquired factor VIII deficiency has been reported to be between 1.48 and 1.34 per million per year in two recent large studies from the UK [1]. Since severe bleeding has been reported to occur in more than 85% of patients and the mortality rate for this condition is very high, ranging from 8% to 22% [1], management of this clinical entity can be challenging.

## Case presentation

**Figure 1:**
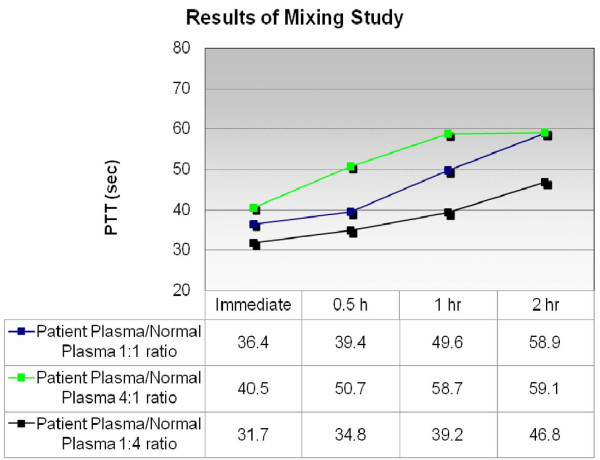
**Results of mixing study using different ratios of patient's plasma or normal plasma at different time points (0, 0.5, 1 and 2 hours)**. Partial thromboplastin time (PTT) is expressed in seconds.

A 94-year-old Caucasian man presented to our hospital with extensive bleeding from his oral cavity and diffuse bruising. His medical history included severe dementia and hypertension. Our patient had a habit of repeatedly biting his tongue. This led to profuse bleeding from the dorsal surface of his tongue that was persistent despite surgical placement of sutures in the tongue and removal of his teeth. His hemostasis was previously normal and he did not take any anticoagulants or non-steroidal anti-inflammatory drugs. There was no nose bleeding, hematuria, bloody stool or accompanying hemoptysis. Our patient did not have any family history of bleeding disorders. On examination, his vital signs were stable and he was afebrile. There was profuse bleeding from the tongue with the presence of multiple clots in the oral cavity. No other bruising or active bleeding was noticed, except extensive bruising over his upper extremities and the presence of a hematoma on the left hand with active oozing. Laboratory tests revealed a white blood cell count of 9500 cells/μL with an initial hemoglobin level of 11.7 g/dL and a platelet count of 149 × 10^3 ^cells/μL. Coagulation studies revealed a normal prothrombin time and international normalized ratio, and a prolonged activated partial thromboplastin time of 73 seconds (normal: 24.8 to 36.1 seconds). The presence of an inhibitor of coagulation was diagnosed via prolonged activated partial thromboplastin time and a mixing study that did not correct with the addition of normal plasma (partial thromboplastin time (PTT) 36.4 seconds when an immediate mixing test was performed, with a ratio of patient's plasma to normal plasma of 1:1) (Figure 1). Quantitative assays revealed a reduced level of factor VIII activity (1%) and the presence of factor VIII inhibitor measured at 7 Bethesda units (BU) in the serum.

Our patient was not intubated for airway protection based on the wishes of his family. In total, two units of fresh frozen plasma and eight units of packed red blood cells were transfused and three doses (100 U/kg) of recombinant human factor VIII (Humate-P 2958 RCO) were given to our patient as initial management. Humate-P was chosen based on the lack of an alternative bypass agent such as recombinant activated FVII (rFVIIa) in the setting of acute severe bleeding. Oral prednisone therapy (60 mg/day) was given, and he also received two doses of intravenous aminocaproic acid (3 g intravenously over 1 hour followed by an infusion of 750 mg/hour for 8 hours) and topical anti-thrombolytic agents (topical thrombin cream 100 units was used once) because of ongoing and active bleeding. His hospital course was also complicated by complete heart block, which developed immediately after the infusion of the second dose of intravenous aminocaproic acid. Of note, our patient was initially admitted with a heart rate of 80 beats/minute and he had a first-degree AV block and left anterior fascicular block on his admission electrocardiogram. Our patient was not a candidate for a transvenous pacemaker secondary to his severe coagulopathy. He required use of vasopressors initially, but he subsequently remained hemodynamically stable with a heart rate of 30 beats/minute. He developed profuse melena for two weeks, most likely as a consequence of swallowing the blood coming from his tongue. His activated partial thromboplastin time (aPTT) gradually improved (38.4 seconds). Our patient's family refused further diagnostic investigation in terms of finding an underlying cause for the acquired hemophilia such as malignancy. He was discharged to a rehabilitation facility with improving symptoms, stable hemoglobin (9 g/dL) and minimized bruises after three weeks of hospitalization. A repeat test for the level of factor VIII inhibitor in serum four weeks after our patient was on steroids showed a reduction to 1BU while VIII activity had also increased (10%). Our patient was discharged on 40 mg of prednisone as immunosuppressive therapy with a treatment plan for a slow tapering of steroids as well as careful monitoring of his coagulation parameters. On follow-up six weeks after discharge, his bradycardia had reversed and his heart rate had increased to 85 beats/minute, which suggests that the initial bradycardia was likely related to the infusion of aminocaproic acid.

## Discussion

Acquired inhibitors against factor VIII, also termed acquired hemophilia A, occurs rarely, with an incidence of approximately 1 to 4 per million/year. Although uncommon, this condition is associated with a high rate of morbidity and mortality as severe bleeding occurs in up to 90% of affected patients [1]. For these reasons, patients with acquired hemophilia A represent a clinical challenge.

The etiology of acquired hemophilia A remains unclear. In approximately half of cases, factor VIII autoantibodies occur in patients without any identifiable cause, while the remaining cases may be associated with autoimmune diseases, infections, use of medications in the post-partum period, or underlying hematological or solid tumors [1]. The diagnosis can be difficult to make and bleeding tends to occur in soft tissue, the retroperitoneal space, and the gastrointestinal and genitourinary tracts [1].

Treatment should be focused on controlling the immediate bleeding episode and suppressing the immune reaction against the coagulant factor. Immunosuppressive therapy with steroids (1 mg/kg/day orally for four to six weeks according to recent guidelines) or cyclophosphamide for inhibitor eradication should begin immediately after diagnosis is made [1,2].

Several different medications are available to control bleeding. Anti-fibrinolytics are increasingly being used to limit blood loss in major surgical procedures and in patients with mucosal bleeding [3]. More specifically, epsilon aminocaproic acid counteracts fibrinolytic activity by reversibly blocking lysine binding sites on plasminogen molecules [3] and has been used mostly in patients undergoing cardiac surgery and orthotropic liver transplantation [3]. Aminocaproic acid is generally well tolerated but adverse events include gastrointestinal reactions, headache, edema, bradycardia, hypotension, thrombosis and rhabdomyolysis [3]. Although aminocaproic acid has been used extensively in congenital hemophilia [4], we describe only the sixth case of use of aminocaproic acid in a setting of acquired hemophilia [4-8]. We found only one other case in the literature of severe bradycardia that developed in the setting of severe bleeding from acquired factor VIII inhibitor, but the authors did not address whether this bradycardia was associated with the infusion of aminocaproic acid [5]. However, immediately after the second infusion of aminocaproic acid our patient developed complete heart block and became hypotensive. However, the contribution of an underlying conduction abnormality cannot be excluded. Placement of a pacemaker was not attempted since this has been associated with severe complications in the setting of acquired factor VIII inhibitors [5].

In patients who have developed antibodies to factor VIII, a number of options are available. In patients with higher titers of inhibitor, activated factor VII can be used [2]. Recombinant activated coagulation FVII (rFVIIa) has recently been licensed for use in acquired hemophilia in the US [2]. By directly activating FX on the surface of activated platelets at the site of injury (thereby bypassing FVIII and FIX), rFVIIa can circumvent the actions of inhibitory antibodies present in patients with acquired hemophilia [2]. Human FVIII concentrates usually represent an inadequate hemostatic therapy unless the inhibitor titer is low (that is, less than 5BU) [2]. Plasma-derived or recombinant human FVIII concentrates can be used in patients with low-titer inhibitors, which should be administered at doses sufficient to overwhelm the inhibitor and thus achieve hemostatic levels of factor VIII [2]. Hemostasis can usually be achieved if plasma levels are raised from 30% to 50% [9,10]. Although Humate-P has been used extensively for treatment of von Willebrand disease, experience with its use in factor VIII inhibitor remains very limited [9,10]. According to recent recommendations, human plasma-derived or recombinant FVIII concentrates can be used in acquired hemophilia for the treatment of minor bleeding manifestations and acute bleeding episodes when the inhibitor titer is low (≤ 5BU) [2], and no bypassing agent is immediately available, as was the case with our patient. Autoantibodies can be very difficult to saturate with factor VIII concentrate due to the variability of inhibitor pharmacokinetics. Although there are no prospective, randomized, controlled clinical studies to assess the dosing of factor VIII concentrate in the setting of acquired hemophilia, according to previous studies, a bolus loading dose of factor concentrate (usually 20 to 50 IU/kg) can be used to neutralize the inhibitor, and for maintenance subsequent doses of factor concentrate can be given either by bolus (20 to 50 IU/kg every 6 to 8 hours) or by continuous infusion (3 to 4 IU/kg/hour) [2]. The Bethesda assay was not immediately available in our case and the lack of another bypass agent in the setting of severe bleeding from the upper airways led us to the decision to administer recombinant factor VIII. We used a relatively high Humate-P dose, and three boluses (100 IU/kg) were given 12 hours apart with adequate hemostasis and progressive control of the bleeding from the tongue. Thus, our case adds to the clinical experience of use of Humate-P in cases of acquired factor VIII deficiency. The dosage of FVIII concentrate should be adjusted depending on plasma FVIII levels and bleeding symptoms [2]. Another interesting finding in our case was the presence of persistent melena for two weeks in the setting of persistent bleeding from the tongue secondary to acquired factor VIII inhibitor. While bleeding from soft tissues and mucosal surfaces has been described in the setting of this coagulopathy, such profound life-threatening bleeding from the tongue has not been described previously, to our knowledge. Our patient responded well to immunosuppression with corticosteroids, and he will remain on tapering doses of corticosteroids with monitoring of factor VIII activity and factor VIII inhibitor levels.

## Conclusions

In conclusion, acquired hemophilia A is an extremely rare clinical entity. Experience with concomitant administration of anti-fibrinolytics and rFVIIIa treatment in patients with this entity is limited. Use of Humate-P can be useful in this coagulopathy, whereas use of aminocaproic acid in states of acquired hemophilia may sometimes be associated with life-threatening complications including bradycardia. Diagnosis of acquired hemophilia requires clinical acumen, and clinicians should suspect a diagnosis of acquired hemophilia in patients with unexplained persistent and profound bleeding from soft tissue and mucosa and in any patient who presents with bleeding and a prolonged activated partial thromboplastin time without other cause.

## Consent

Written informed consent was obtained from the patient's next of kin for publication of this case report and any accompanying images. A copy of the written consent is available for review by the Editor-in-Chief of this journal.

## Competing interests

The authors declare that they have no competing interests.

## Authors' contributions

TK analyzed and interpreted the patient data and was a major contributor in writing the manuscript. JR analyzed the patient data and contributed in writing the manuscript. RP and BE analyzed and interpreted the patient data and were major contributors in writing the manuscript. All authors read and approved the final manuscript.
