# Correction:Acquired hemophilia as the cause of life-threatening hemorrhage in a 94-year-old man: a case report

**DOI:** 10.1186/1752-1947-5-73

**Published:** 2011-02-22

**Authors:** Theodoros Kelesidis, Jonelle Raphael, Elizabeth Blanchard

**Affiliations:** 1 Department of Medicine, Caritas St Elizabeth's Medical Center, Tufts University School of Medicine, Boston, MA, USA

## Correction

The list of authors of this article [1] was incorrect as published and should be as follows: Theodoros Kelesidis, Jonelle Raphael and Elizabeth Blanchard.

Rekha Parameswaran did not approve the final version of the manuscript for publication and does not wish to be included as an author. In addition, Elizabeth Blanchard's initials appeared incorrectly as BE in the authors' contributions section. This section should read as follows:

TK analyzed and interpreted the patient data and was a major contributor in writing the manuscript. JR analyzed the patient data and contributed in writing the manuscript. EB analyzed and interpreted the patient data and was a major contributor in writing the manuscript. All authors read and approved the final manuscript.
